# Supplementary material for: Genome Resolved Biogeography of Mamiellales
Source: Genes (Basel). 2020 Jan 7;11(1):66. doi: 10.3390/genes11010066 (PMC7016971; doi:10.3390/genes11010066)
Supplement: Supplementary file 1 [file genes-11-00066-s001.zip › Supplementary_legends.docx]

**Supplementary Materials:**

**Table S1.** Metagenomic samples identifying information.

**Table S2.** Post-hoc Tukey test pairwise pvalues for each of the 4 significant environmental parameters according to the Kruskal-wallis test.

**Table S3.** Relative metagenomic abundances for the six Mamiellales genomes in all samples.

**Figure S1.** Metagenomic reads coverage along chromosome 2 of *O. lucimarinus* in the 11 samples where this genome recruits at least 0.1% of reads.

**Figure S2.** Scatterplot of *O. lucimarinus* chromosome 2 differential coverage based on median values against the ratio of relative metagenomic abundances of the *MT-* and *MT+* mating types genes.

**Figure S3.** Barplot comparisons of relative metagenomic abundances of the *MT-* and *MT+* mating types (left panel) versus whole genome (right panel) for *O. lucimarinus*
