# Supplementary figures and images for: Genome Resolved Biogeography of Mamiellales
Source: Genes (Basel). 2020 Jan 7;11(1):66. doi: 10.3390/genes11010066 (PMC7016971; doi:10.3390/genes11010066)

Coverage

6DCM

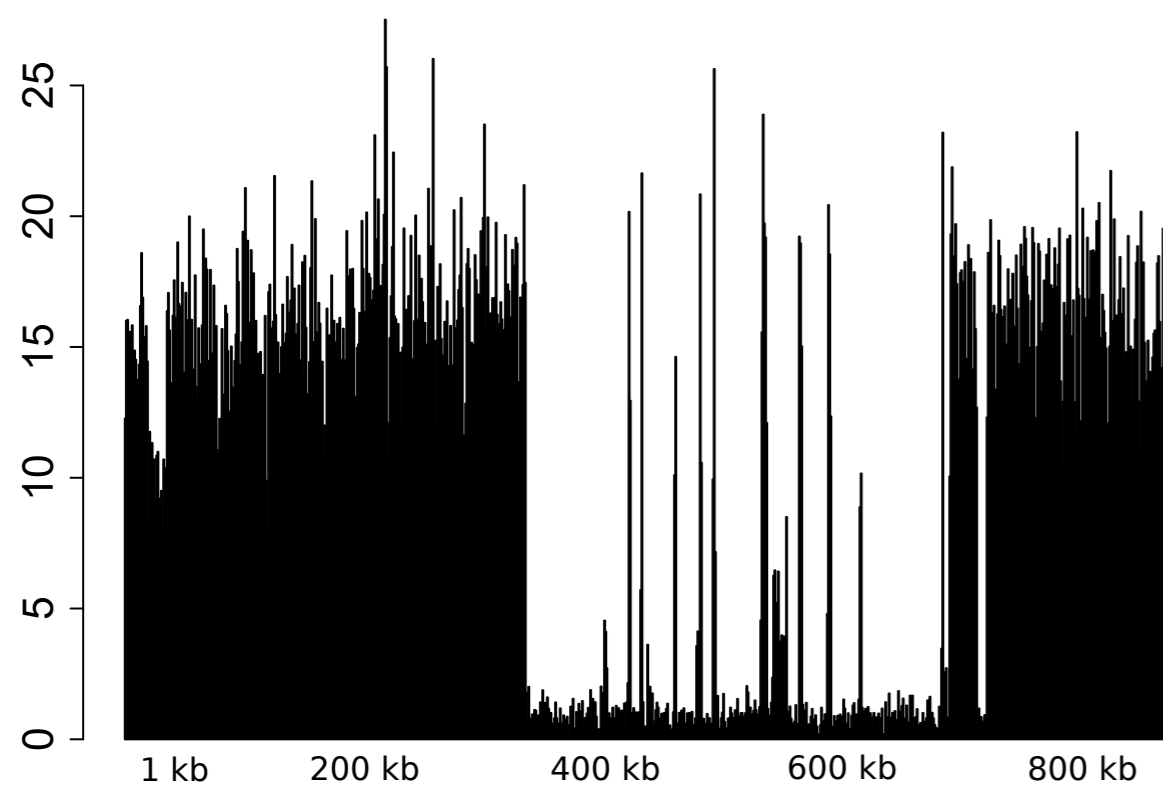

6SUR

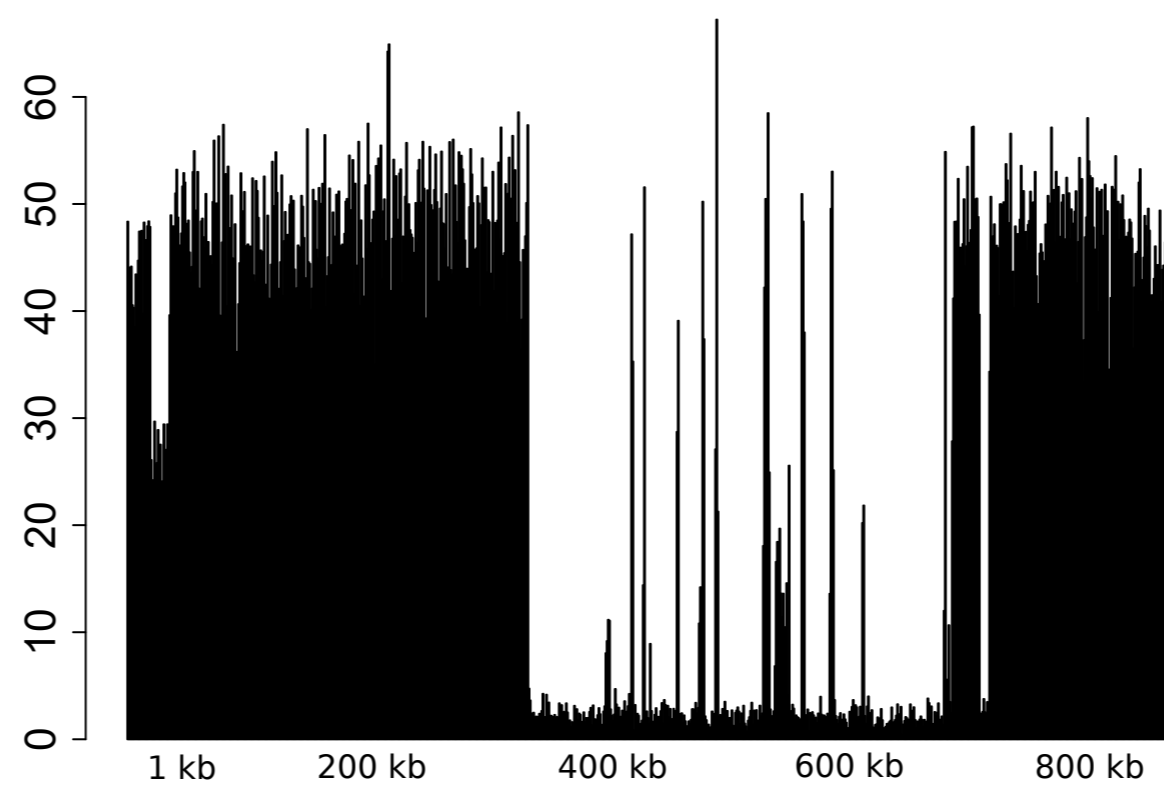

7DCM

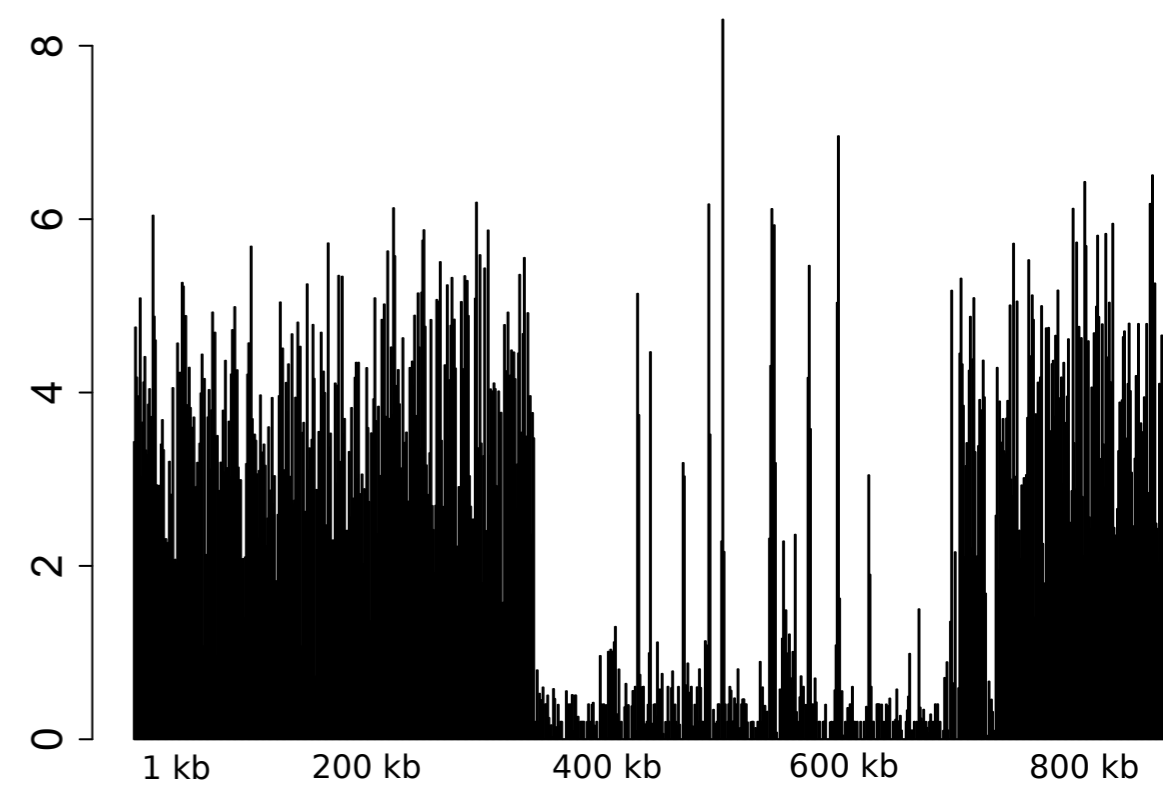

66DCM

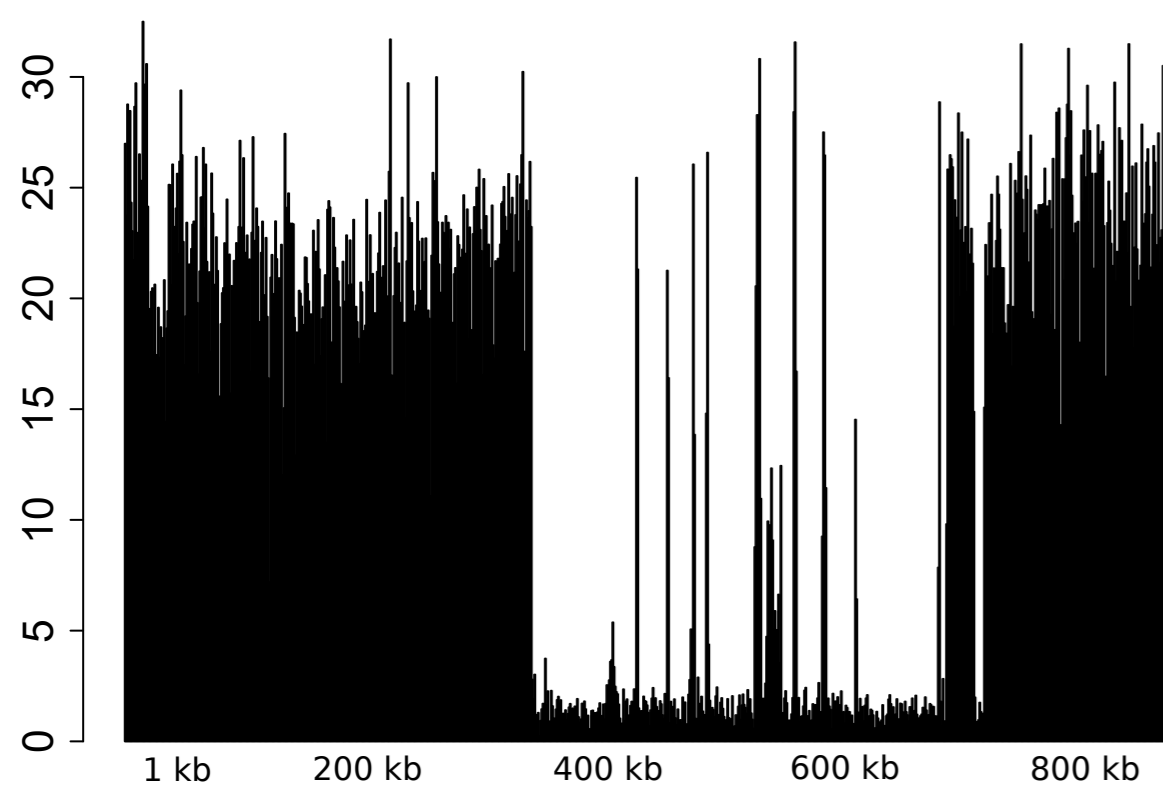

66SUR

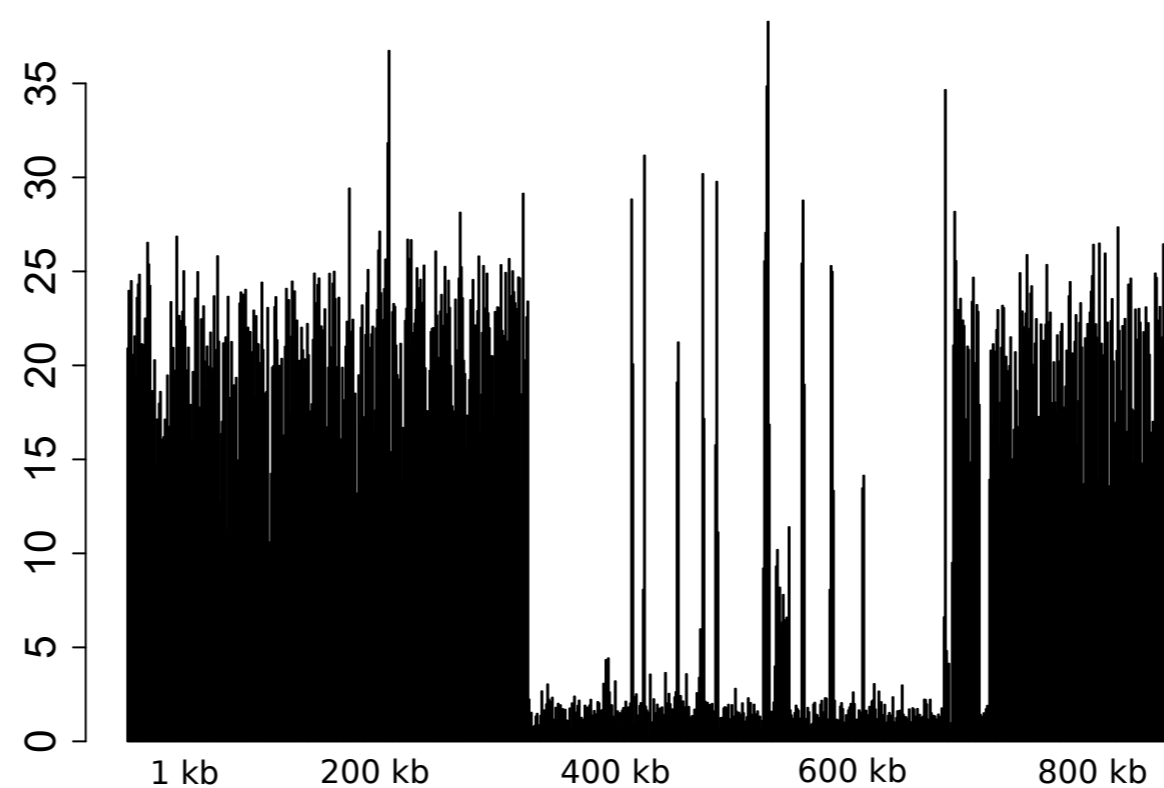

67SUR

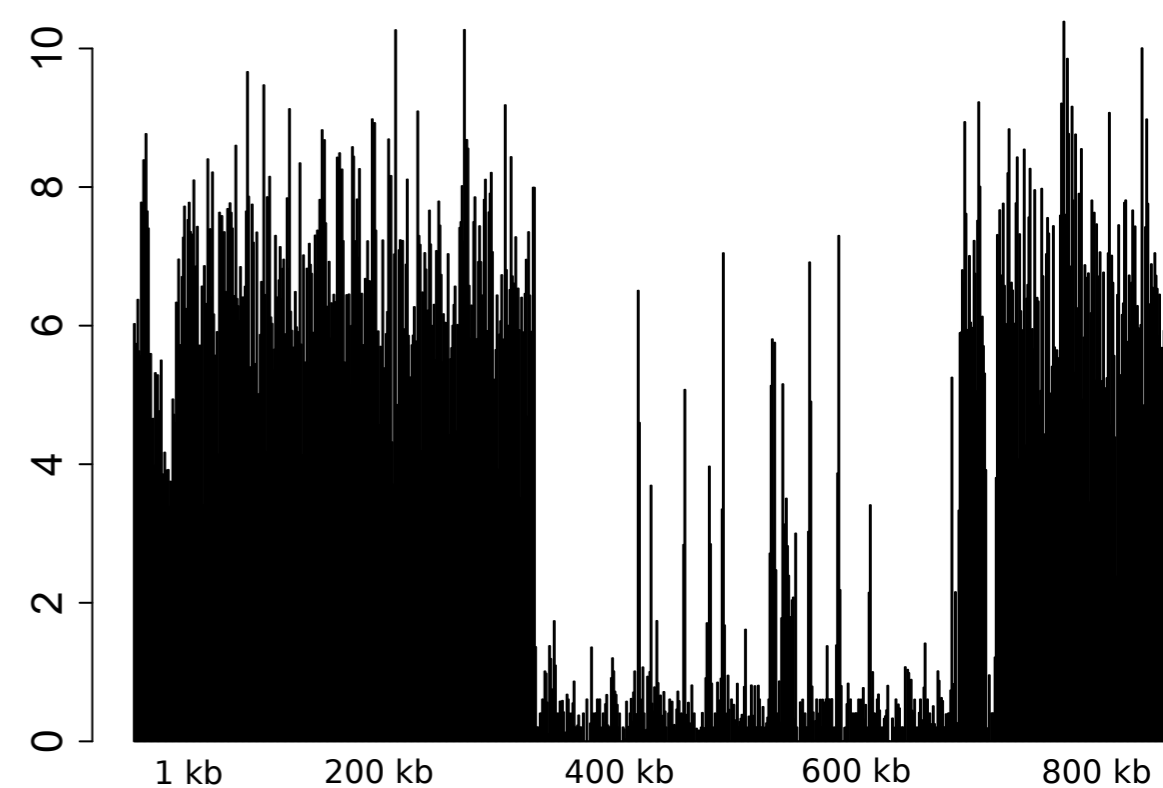

81DCM

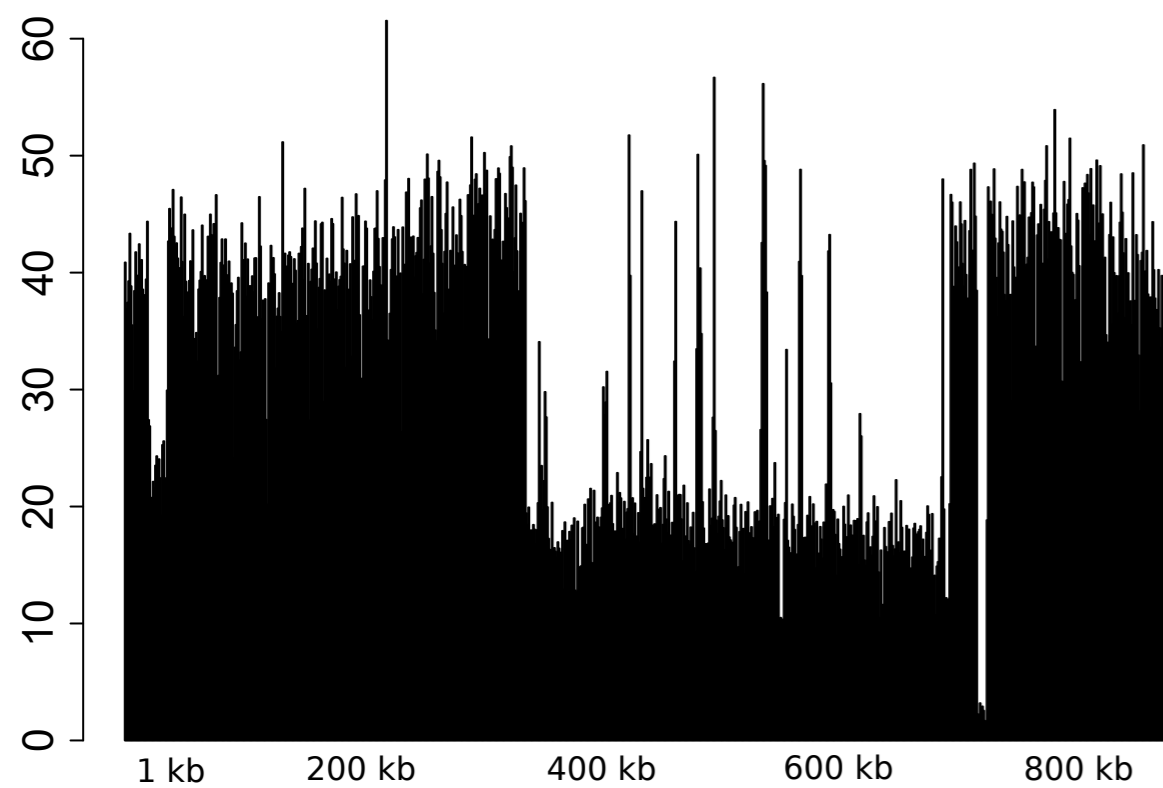

81SUR

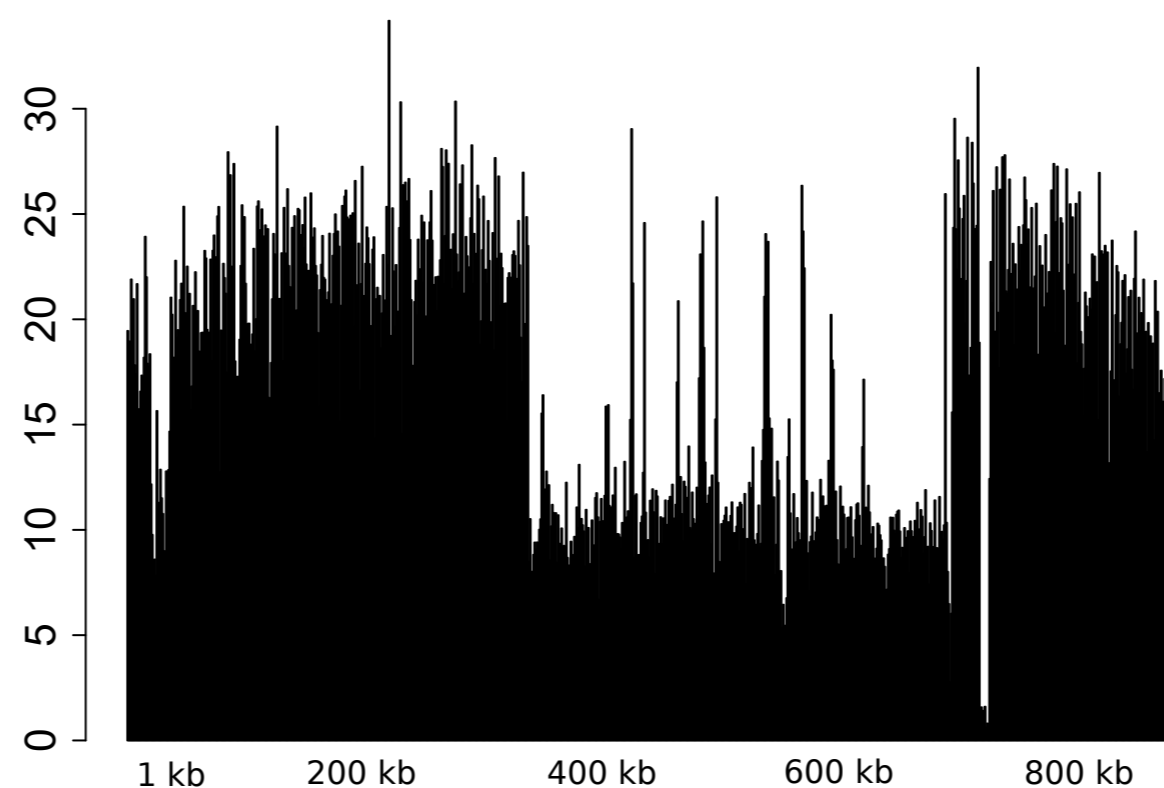

92SUR

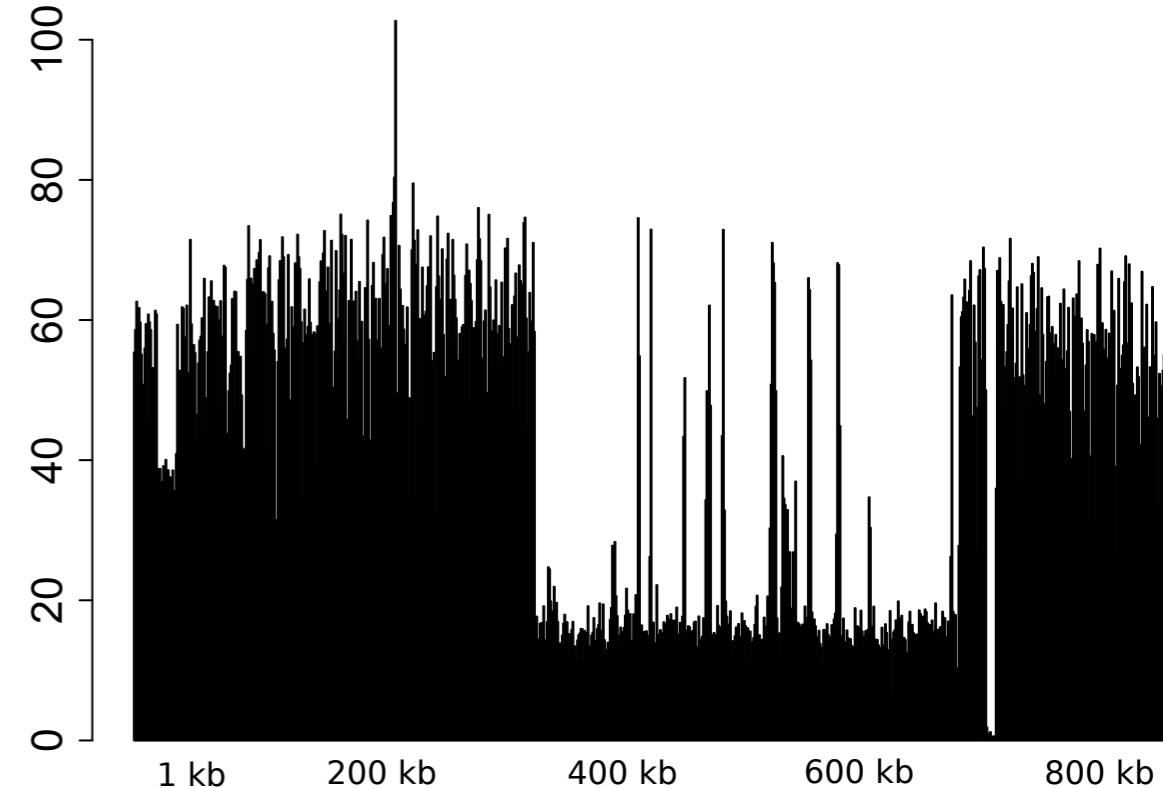

135DCM

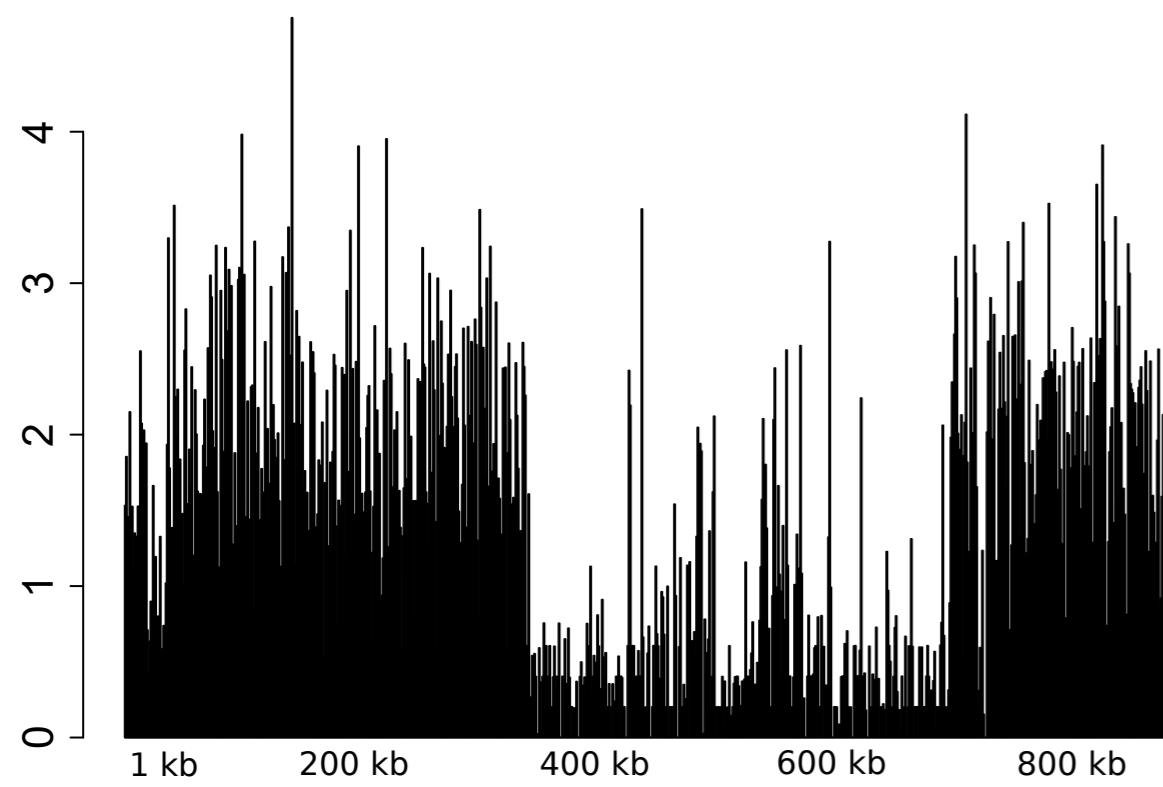

145SUR

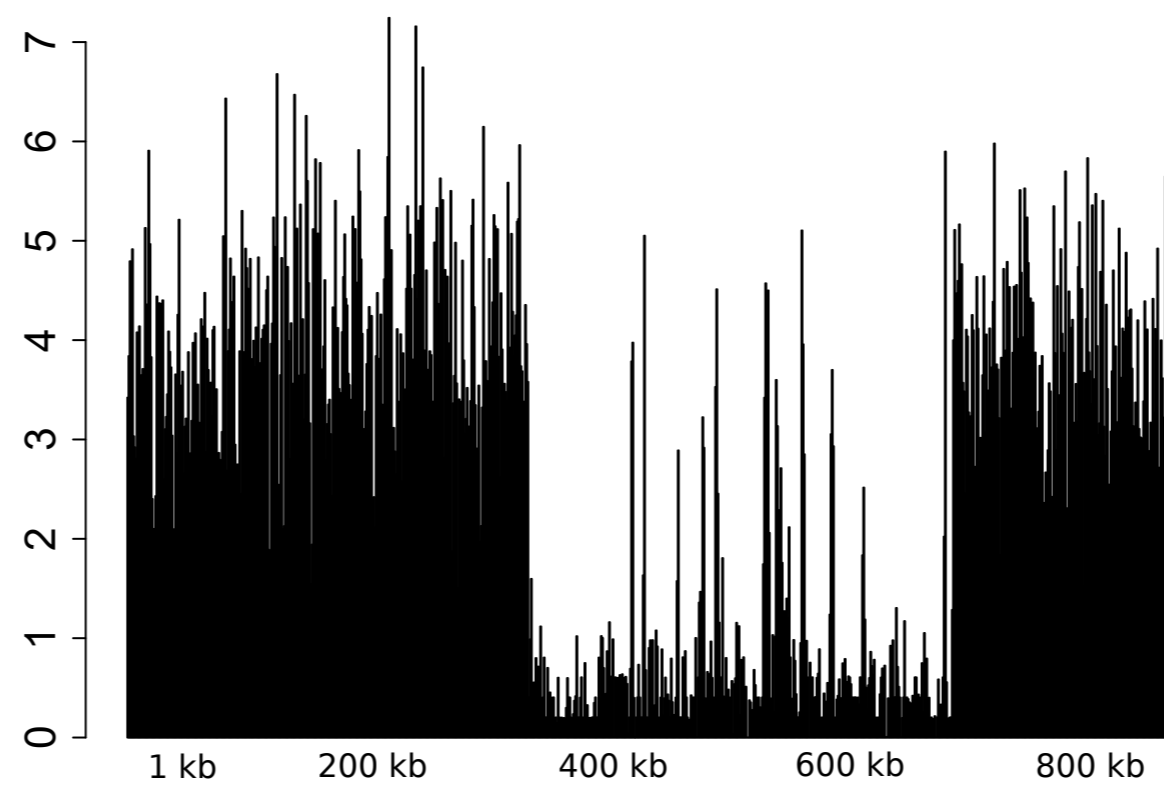

Chromosome 2 position

Supplement: Supplementary file 1 [file genes-11-00066-s001.zip › Figure S1.pdf]

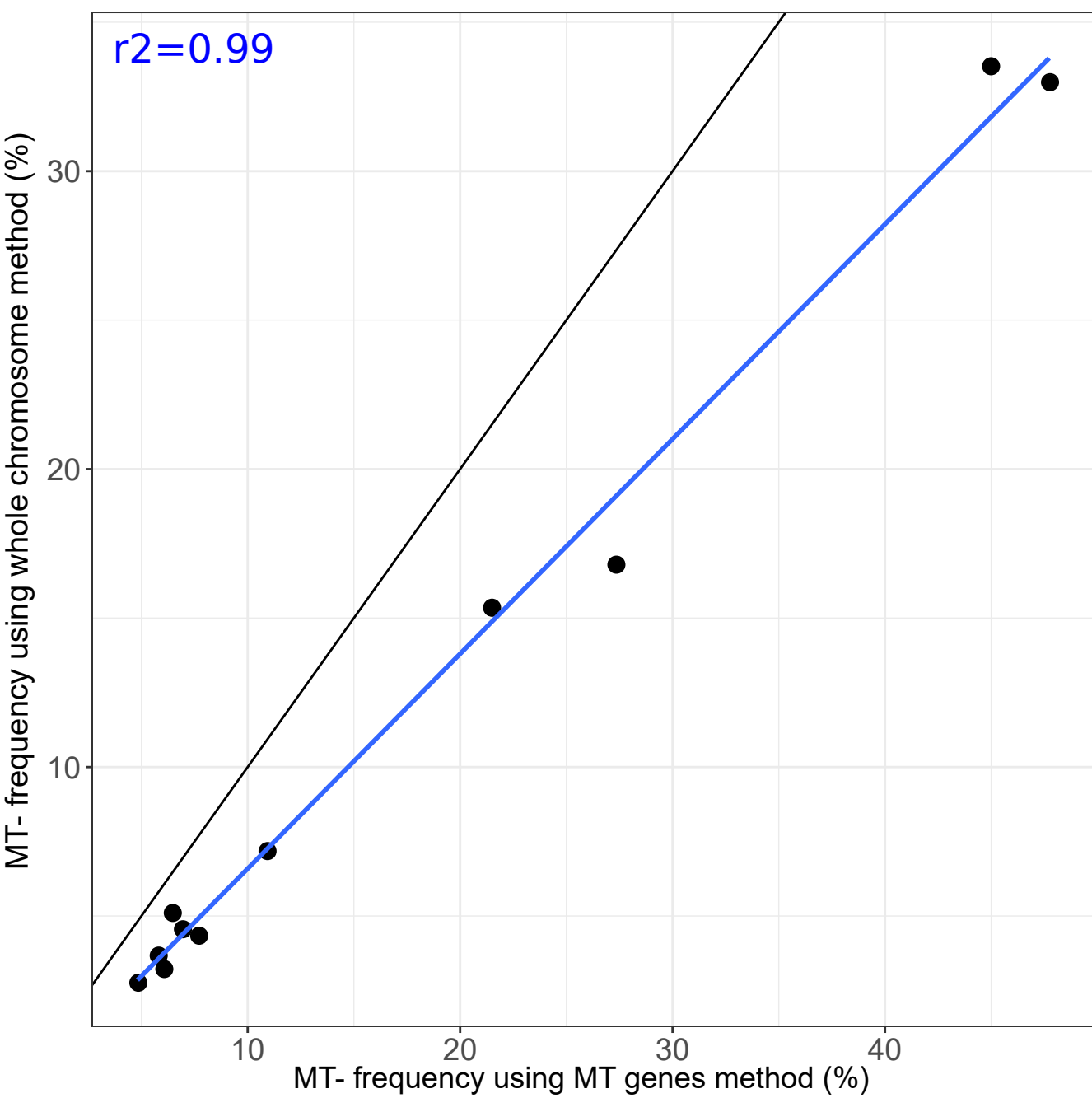

Supplement: Supplementary file 1 [file genes-11-00066-s001.zip › Figure S2.pdf]

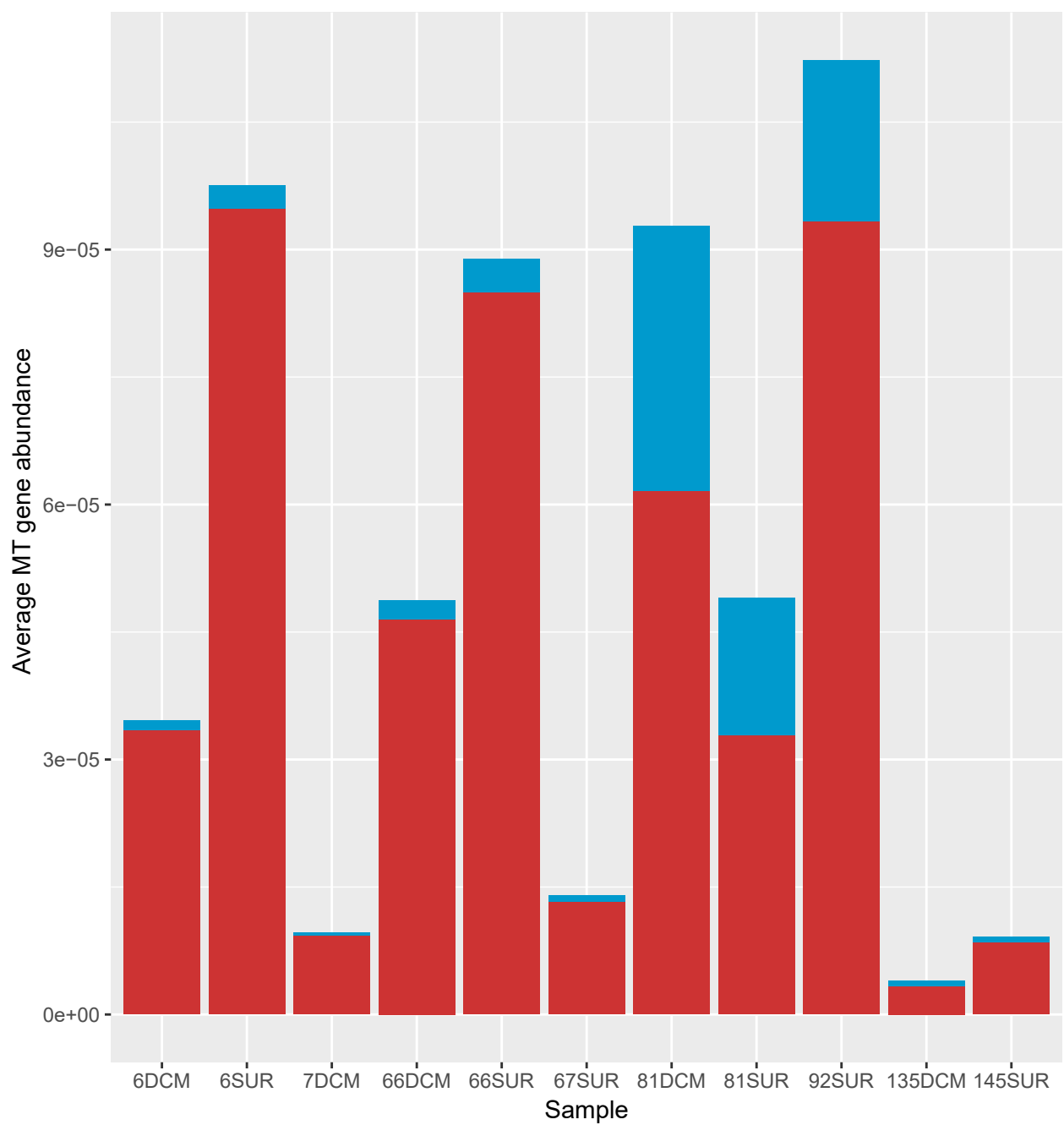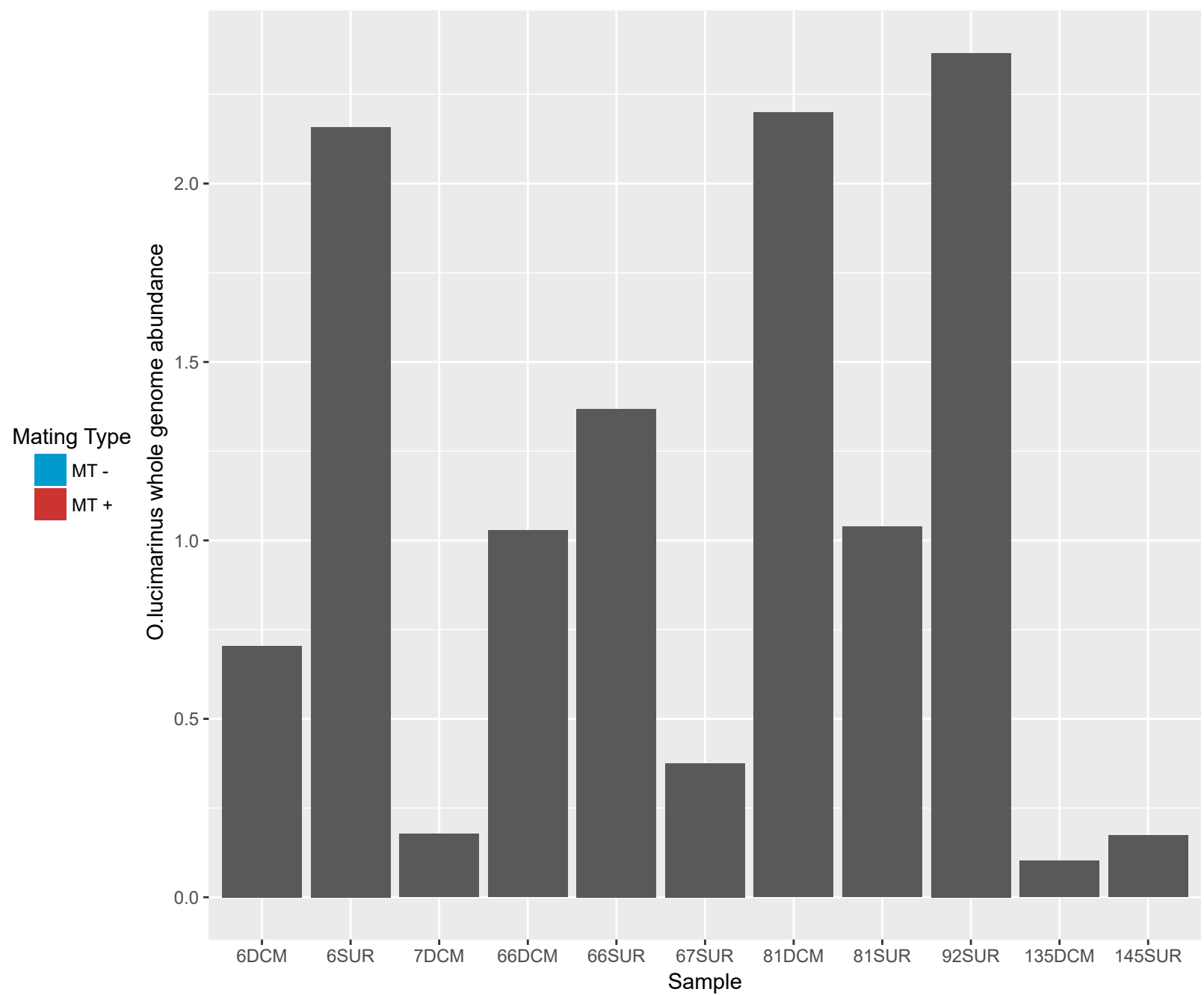

Supplement: Supplementary file 1 [file genes-11-00066-s001.zip › Figure S3.pdf]
